# An amine oxidase gene from mud crab, *Scylla paramamosain*, regulates the neurotransmitters serotonin and dopamine *in vitro*

**DOI:** 10.1371/journal.pone.0204325

**Published:** 2018-09-24

**Authors:** Junguo Liu, Ming Zhao, Wei Song, Lingbo Ma, Xiu Li, Fengying Zhang, Le Diao, Yan Pi, Keji Jiang

**Affiliations:** 1 Key Laboratory of Aquatic Genomics, Oceanic and Polar Fisheries, Ministry of Agriculture and Rural Affairs, East China Sea Fisheries Research Institute, Chinese Academy of Fishery Sciences, Shanghai, China; 2 College of Fisheries and Life Sciences, Shanghai Ocean University, Shanghai, China; 3 School of Life Sciences, Fudan University, Shanghai, China; Chinese Academy of Sciences, CHINA

## Abstract

Amine oxidase, which participates in the metabolic processing of biogenic amines, is widely found in organisms, including higher organisms and various microorganisms. In this study, the full-length cDNA of a novel amine oxidase gene was cloned from the mud crab, *Scylla paramamosain*, and termed *SpAMO*. The cDNA sequence was 2,599 bp in length, including an open reading frame of 1,521 bp encoding 506 amino acids. Two amino acid sequence motifs, a flavin adenine dinucleotide-binding domain and a flavin-containing amine oxidoreductase, were highly conserved in *SpAMO*. A quantitative real-time polymerase chain reaction analysis showed that the expression level of *SpAMO* after quercetin treatment was time- and concentration-dependent. The expression of *SpAMO* tended to decrease and then increase in the brain and haemolymph after treatment with 5 mg/kg/d quercetin; after treatment with 50 mg/kg/d quercetin, the expression of *SpAMO* declined rapidly and remained low in the brain and haemolymph. These results indicated that quercetin could inhibit the transcription of *SpAMO*, and the high dose (50 mg/kg/d) had a relatively significant inhibitory effect. *SpAMO* showed the highest catalytic activity on serotonin, followed by dopamine, β-phenylethylamine, and spermine, suggesting that the specific substrates of *SpAMO* are serotonin and dopamine. A bioinformatics analysis of *SpAMO* showed that it has molecular characteristics of spermine oxidase, but a quercetin test and enzyme activity study indicated that it also functions like monoamine oxidase. It is speculated that *SpAMO* might be a novel amine oxidase in *S*. *paramamosain* that has the functions of both spermine oxidase and monoamine oxidase.

## 1. Introduction

Mud crab, *Scylla paramamosain* are economically important crustaceans cultured in brackish coastal waters of the Southwest Pacific Ocean and North Indian Ocean [1]. The total output is more than 110,000 tons per year, but the supply of seeds mainly depends on catching them from the wild, as cultivated seeds cannot meet quantity and quality requirements [2]. Cannibalism is deemed to be one of the limited factors for aquaculture expansion. The more cannibalistic, the higher-value species they’ve got [3–5]. The crabs’ aggressive behaviour is a bottleneck problem during crab culturing and has caused severe economic losses. Molt is crucial in development and reproduction of crustaceans. This can be deduced from two main aspects: crustaceans are highly vulnerable to cannibalism during the molting process because their new shell is incompletely calcified; the potential victim can then become the attacker on smaller conspecifics after successful molting because substantial size increases [6–7]. There are six metamorphoses from the zoea I to the juvenile crab I stage, and cannibalism among individuals from the megalopa stage to the juvenile crab I stage often leads to massive die-offs. The traditional breeding method can only reduce the cannibalize rate by increasing bait quantity, reducing the crab density and setting up shelters, among other measures. There are few studies on the crabs’ aggressive behaviour; we hope to discover the genes related to aggressive behaviour in *S*. *paramamosain*, and to explore the molecular foundation behind the aggressive behaviour.

Amine oxidases catalyse the oxidative deamination of aliphatic monoamines and aromatic amines [8]. These enzymes are classified into two classes based on their prosthetic groups: copper/topaquinone-containing amine oxidases (EC 1.4.3.6) and flavin-containing amine oxidases (EC 1.4.3.4) [9]. The main copper-containing amine oxidases are primary amine oxidase and diamine oxidase, which are widely found in nature [10–11]. The main flavin-containing amine oxidase is monoamine oxidase (MAO), which is found in most living things. Spermine oxidase (SMO), which has recently been successfully cloned and identified, is primarily involved in polyamine degradation but is also a flavoprotein [12]. Polyamines (such as putrescine, spermidine, and spermine) are small organic molecules that are essential for the growth and differentiation of mammalian cells and that widely participate in chromosomal formation, gene transcription activation, signal transduction, apoptosis and other important physiological and pathological processes [13–14]. MAO is a flavoenzyme in which the flavin adenine dinucleotide (FAD) is covalently anchored to a cysteine residue by an 8α-(S-cysteinyl)-riboflavin linkage [15]. According to previous studies [16–17], MAO is usually anchored to the mitochondrial outer membrane of neuronal, glial and several other cell types, and it can catalyse the oxidative deamination of biogenic and xenobiotic amines into aldehydes and ammonia in the peripheral and the central nervous system. In mammals, MAO exists in two isoforms (MAO-A and MAO-B) that are dimeric in their membrane-bound forms [18]. By cloning MAO cDNA, it was subsequently demonstrated that MAO-A and MAO-B were two distinct proteins with a high degree of sequence identity, encoded by different genes having identical exon-intron organization and regulated by different gene regulators [19–20]. The X-ray crystal structures of the two human isoforms show that the active site amino acid residues and their relative geometries are highly conserved, and only six of the 16 active site residues differ between the two isozymes [16,21–22]. Despite their similarities, MAO-A and MAO-B differ in their tissue distributions and substrate/inhibitor specificities. MAO-A catalyses the deamination of serotonin (5-hydroxytryptamine, 5-HT), adrenaline and noradrenaline and is selectively inhibited by clorgyline, moclobemide and quercetin; in contrast, MAO-B catalyses the deamination of β-phenylethylamine (PEA) and benzylamine and is irreversibly inhibited by selegiline [23]. It is interesting that both dopamine (DA) and tyramine are deaminated by the two isoforms *in vitro*, but dopamine is primarily metabolized by MAO-B in the human body. Because of their ability to promote the catabolism of neurotransmitter amines, MAO-A and MAO-B are considered attractive drug targets in therapies for neurological disorders. For example, MAO-A inhibitors are prescribed for the treatment of depression and anxiety disorders, and MAO-B inhibitors are used as L-DOPA and/or dopamine agonists in the symptomatic treatment of Parkinson’s disease.

Biogenic amines can transmit various information as neurotransmitters or hormones, and participate in physiological processes of osmoregulation [24–25], behavioral regulation [26], immunological stress [27], and neuroendocrine [28] in crustaceans. DA is a kind of biogenic amine which has the effect of gonadotropin releasing factor [29]. Chen et al. [30] speculated that DA may participate in pathological aggressive behavior and other abnormal behaviors. DA synthesis, degradation, and transitive processes may affect each individual's behavior [31]. 5-HT, also known as serotonin, is an inhibitory neurotransmitter in biogenic amines. 5-HT is the most important neurotransmitter affecting aggressive behavior [32–34]. Other neurotransmitters may work by first affecting 5-HT [35]. Wei et al. [36] and Kravitz [26] considered that 5-HT was an important monoamine neurotransmitter and was closely related to the fighting behavior of aquatic animals. Zhao et al. [37] compared with effects of injection of DA and 5-HT on the fighting behavior of *Penaeus japonicus*, *Litopenaeus vannamei* and *Fenneropenaeus chinensis*, and found that the average number of fight of three kinds of shrimps significantly increased after being injected with dopamine, and high DA was more likely to stimulate competition among shrimps than low DA. However, the average number of fighting shrimps decreased with the 5-HT concentration increasing. Similar conclusion appeared in crayfish *Procambarus clarkii*. When crayfish were placed in a space-limited aquarium, there was constantly fighting behavior until one side was defeated and clearly expressed a submissive behavior. These findings suggested the role of 5-HT, whose brain concentrations increased much more in losers than in winners [38]. As a key enzyme in the 5-HT and DA degradation pathways, MAO-A plays an important role in emotional regulation and is related to the severity of aggressive and antisocial traits [39–42]. For example, Brunner syndrome, a genetic condition characterized by a nonsense point mutation in the MAO-A gene, results in marked increases in urinary 5-HT levels, antisocial behaviour, reactive aggression and mild cognitive impairment [41]. While there has been considerable research on MAO in mammals and other vertebrates, such as reptiles [43], birds [44] and teleosts [45–46], there are no reports on MAO in crustaceans. Furthermore, although research has shown that MAO-A deficiency is related to reactive aggression in humans [41,47] and mice [42,48], the relationship between this enzyme and aggressive behaviour in crabs is still unclear. This study seeks to characterize the SpAMO gene in *S*. *paramamosain* and determine its biological function and its relationship with MAO-A.

## 2. Materials and methods

### 2.1 Ethics statement

All animal experiments in this study were conducted in accordance with the relevant national and international guidelines. Our project was approved by the East China Sea Fisheries Research Institute. In China, catching wild mud crabs from seawater does not require specific permits. Our study did not involve endangered or protected species.

### 2.2 Materials, reagents and total RNA extraction

Mud crabs, with uniform size (200±20 g), well-characterized and good vibrancy, were collected from Hainan Island, China. Different organs and tissues, including thoracic ganglia, brain and haemolymph, were immediately collected and preserved in liquid nitrogen for RNA extraction. Three biological replicated samples from different individuals were collected. Total RNA was isolated from the thoracic ganglia using Unizol reagent (Biostar, Shanghai, China) following the manufacturer’s instructions. The total RNA was treated with RNase-free DNase I (Sigma, St. Louis, USA) to eliminate possible contamination by genomic DNA and then stored at -80 °C for follow-up experiments. The quality and concentration were checked by agarose gel electrophoresis and spectrophotometry (DU 800, Beckman Coulter, USA).

First-strand cDNA synthesis was performed from poly(A) mRNA template using M-MLV reverse transcriptase (Promega Corporation, Madison, WI, USA) with Oligo-dT and random 6-mer primers. The reaction conditions were as recommended by the manufacturer.

### 2.3 cDNA library construction and cloning of full-length *SpAMO* cDNA

The *S*. *paramamosain* cDNA library was successfully constructed by using a SMART^TM^ cDNA Library Construction Kit (Clontech, Palo Alto, CA, USA), and all the EST sequences were subjected to BLAST analysis. BLAST analysis showed that one EST sequence was highly similar to the previously identified amine oxidases, and therefore that sequence was selected for cloning of *SpAMO* cDNA.

A fragment of *SpAMO* was identified in the cDNA library constructed in our laboratory from the *S*. *paramamosain* thoracic ganglia. The full-length *SpAMO* cDNA was obtained by the rapid amplification of cDNA ends (RACE) method, which was performed using a SMART^TM^ RACE cDNA Amplification Kit (Clontech) according to the manufacturer’s instructions. The primers for 3’ and 5’ RACE were termed SpAMO-3’race (5’-TCGCCGTGAAGTGGAGGT-3’) and SpAMO-5’race (5’-ACGAGTGTTGTGGGTATGCT-3’), respectively. The PCR fragments were analysed by electrophoresis on 1.5% agarose gels to determine length differences. Amplified cDNA fragments were cloned into the pMD18-T vector (TaKaRa, Beijing, China) following the manufacturer’s instructions. Recombinant bacteria were identified by blue/white screening and confirmed by PCR. Plasmids containing the inserted *SpAMO* fragment were used as templates for DNA sequencing.

### 2.4 Sequence analysis of *SpAMO*

The identity searches for the nucleotide and protein sequences were performed using the BLAST algorithm of NCBI (http://www.ncbi.nlm.nih.gov/). The deduced amino acid sequence was analysed with the Expert Protein Analysis System (http://www.expasy.org/). Amino acid sequences from various species were retrieved from the NCBI GenBank database and analysed using the Vector NTI Suite 11.0 and the Clustal W multiple alignment program (http://www.ebi.ac.uk/clustalw). A neighbour-joining phylogenetic tree was constructed using MEGA 5.1, and the confidence level in the tree was generated using 1000 bootstraps. The secondary structure was predicted by the application of a hierarchical neural network (http://www.expasy.org/). The 3D structure of *SpAMO* was simulated using the SWISS-MODEL long-distance server.

### 2.5 Expression analysis of *SpAMO* under quercetin treatment

Mud crabs, with uniform size (300±20 g), well-characterized and good vibrancy, were collected from Qionghai, Hainan province, China. Sixty crabs (female-male ratio: 1:1) were temporarily kept in cement pools to acclimate for seven days before experiments under the same environmental conditions. Two gradients of quercetin, i.e., 5 and 50 mg/kg/d were set up in this experiment, and the two experimental groups were placed under the same experimental conditions of dissolved oxygen, light and feeding. The experimental period was 5 days, and the experimental water was changed every day. Both experimental groups had the same trend of temperature (26–30°C) and salinity (27–30 ppt) during the experimental period. In order to keep the concentrations of quercetin at the initial level, corresponding concentrations were injected daily, as required. Then, 24 h after injection, hemolymph and brain tissues were collected at 0h, 24h, 3d, and 5d for analysis of the expression as a response towards quercetin. Three biological replicated samples from different individuals were collected. It should be noted that the tissues sampled at 0 h were not injected with quercetin *in vivo*. The other individuals were injected with quercetin *in vivo*. RNAfixer RNA stabilization reagent (Biosharp, Hefei, China) was added to the samples of haemolymph and brain, and samples were stored at -20 °C [49].

Quantitative real-time polymerase chain reaction (qRT-PCR) was performed to study the expression of *SpAMO* in *S*. *paramamosain*. Total RNA (1 μg) was reverse transcribed into first-strand cDNA with the ReverTra Ace qPCR RT Kit (Toyobo Co., Ltd., Osaka, Japan). A pair of 18S rRNA primers, 18s-RT-F (5’-GGGGTTTGCAATTGTCTCCC-3’) and 18s-RT-R (5’-GGTGTGTACAAAGGGCAGGG-3’), were used to amplify the internal control. Expression levels were calculated by the standard curve method [50]. A SYBR Green qRT-PCR assay (Power SYBR Green PCR Master Mix, Applied Biosystems, Foster City, CA, USA) was carried out in an ABI StepOnePlus detection system (Applied Biosystems). Amplifications were performed in a 96-well plate with a 20 μL reaction volume containing 10 μL of SYBR Premix Ex Taq, 0.8 μL of PCR forward primer (10 mM), 0.8 μL of PCR reverse primer (10 mM), 0.4 μL of ROX reference dye, 2.0 μL of cDNA template and 6.0 μL of diethylpyrocarbonate (DEPC)-treated water. The cycling parameters for SYBR Green qRT-PCR were 10 min at 94 °C; 45 cycles of 94 °C for 15 s and 60 °C for 1 min; and a final elongation at 72 °C for 10 min. Primers used to amplify *SpAMO* were SpAMO-RT-F (5’-GACCAAGCCACCCTCAATCAGT-3’) and SpAMO-RT-R (5’- CGGCGTTTAGGCGGAATAG -3’).

### 2.6 Determination of *SpAMO* activity

To construct the prokaryotic vector plasmid *SpAMO/pCold I*, the target fragment *SpAMO* was first amplified by PCR. The PCR products were then digested by the enzymes *Kpn*I and *Eco*RI (5’-AGGCATATGGAGCTCGGTACCAACACACGTCACCCTTTCGT-3’, 5’-CAGGTCGACAAGCTTGAATTCTCACGGCGAGGATGGCTTGA-3’; the enzyme cleavage sites of *Kpn*I and *Eco*RI, respectively, are in the boxes) and cloned into the cold shock carrier pCold I digested by the same enzymes. The prokaryotic expression plasmid *SpAMO/pCold I* was transformed into BL21 (DE3) competent *Escherichia coli* cells and then cultured in small tubes of fluid. SOC liquid medium (containing ampicillin) was inoculated with the culture medium and incubated at 37 °C for 1–3 h until the OD600 reached 0.4–0.6. IPTG (isopropyl β-D-thiogalactoside) was then added to a final concentration of 0.5 mM, and culturing was continued at 15 °C for 24 h in a shaker. The bacterial strains were collected by centrifugation (~2800×*g* for 10 min), and then the supernatant was collected by ultrasonic crushing treatment. The recombinant protein *SpAMO* contained in the supernatant was separated and purified by Ni-NTA resin affinity chromatography. The purified recombinant protein eluted from the chromatographic column was dialysed, and the urea was gradually removed to restore protein activity. The dialysis buffer was a mixture of 4 M urea buffer (100 mM NaH_2_PO_4_, 10 mM Tris, and 4 M urea, pH 6.0), refolding buffer (20 mM Tris, pH 7.0, containing 2 mM GSH, 0.2 mM GSSG, 5 mM EDTA and 50 mM glycine) and 2 M urea buffer (100 mM NaH_2_PO_4_, 10 mM Tris, and 2 M urea, pH 8.0). The purified protein samples were stored at -80 °C.

The activity of *SpAMO* was determined by the chemiluminescence method [51]. The quantity of active H_2_O_2_ produced by *SpAMO* in the process of oxidizing substrate monoamines was used to calculate enzyme activity. Glycine buffer (50 μL of 0.5 mol/L buffer, pH 8.0), 50 μL of horseradish peroxidase (0.4 g/L), 50 μL of luminol (100 μmol/L), 1 μL of *SpAMO*, and 99 μL of water were added to the test tube (*SpAMO* was replaced with dialysate in the control group), and the samples were incubated at 37 °C for 2 min. Then, 50 μL of serotonin, dopamine, phenylethylamine, or spermine (1.5 mmol/L) was added to the test tube to measure fluorescent counting every 20 s with a Monolight 3010 fluorescence spectrometer. Enzyme activity was measured in pmol H_2_O_2_/mg protein·min.

### 2.7 Statistical analysis

Measurements were performed under quercetin treatment and enzyme assay to derive the mean and standard deviation of testing results. All data obtained from the qRT-PCR and enzyme assay analysis were log transformed before being subjected to data analysis with oneway ANOVA. Differences were considered significant at *P* < 0.05.

## 3. Results

### 3.1 Sequence analysis

**Fig 1:**
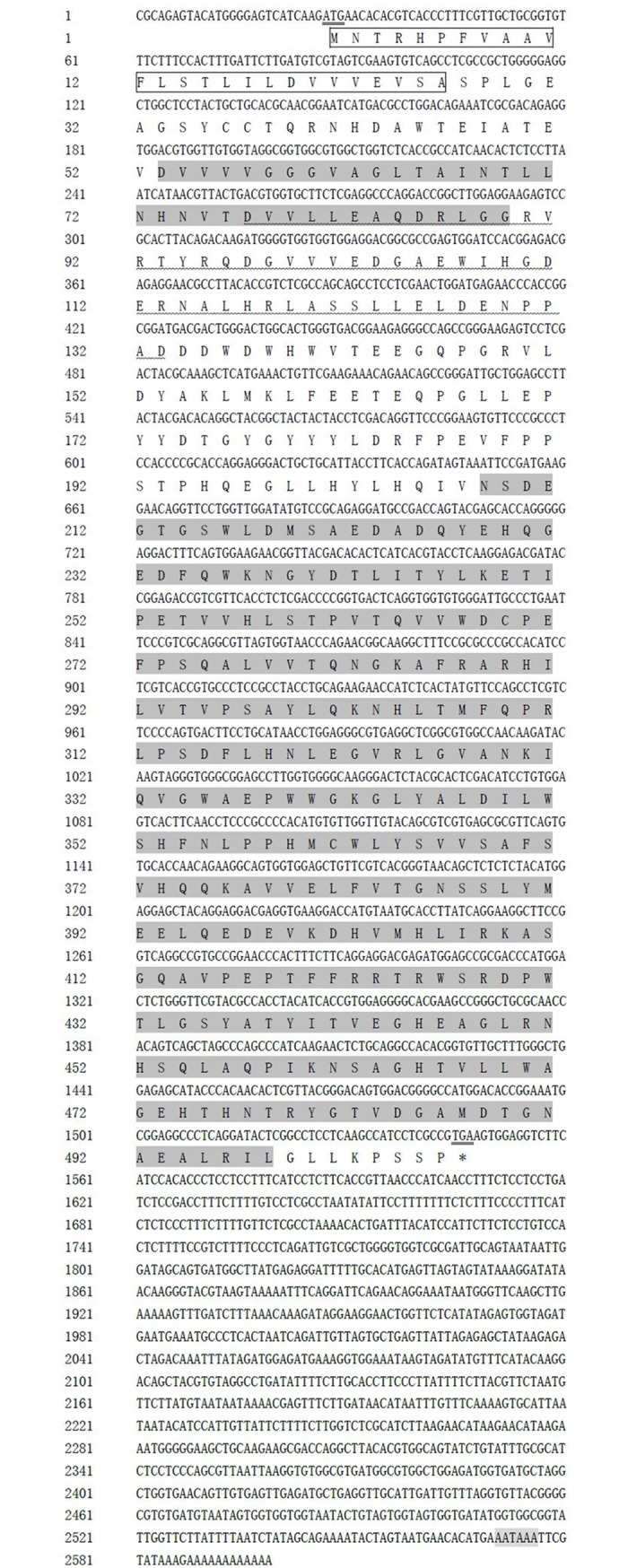
The nucleotide and deduced amino acid sequences of the *SpAMO* gene in *Scylla paramamosain*. Shown are the coding region and parts of the untranslated regions, where the upper sequence is the nucleotide sequence and the lower sequence is the amino acid sequence. The start (ATG) and stop (TGA) codons are double underlined. The parts in shadow indicate the FAD-binding domain (53–89 aa), the flavin-containing amine oxidoreductase (208–498 aa) and the termination signal AATAAA. The heavy line indicates the putative conserved motif proto_IX_ox (77–133 aa). The signal peptide is boxed.

The full-length cDNA fragment of *SpAMO* was 2,599 bp (GenBank accession No. MG878093), and it contained an open reading frame (ORF) of 1,521 bp with a 26 bp 5’ untranslated region (5’-UTR) and a 1,052 bp 3’ untranslated region (3’-UTR). Multiple consensus polyadenylation signals (AATAAA) were found 11 bp upstream of the poly (A) tails. Multiple ATTTA (G) motifs, which are correlated with transcript stability, were found in the 3' UTR region of the *SpAMO*. The ORF encodes a putative protein of 506 amino acids (aa) with a predicted molecular weight of 56.96 kDa and a theoretical isoelectric point of 5.04. The full-length nucleotide sequence and the deduced amino acid sequence are shown in Fig 1.

**Fig 2:**
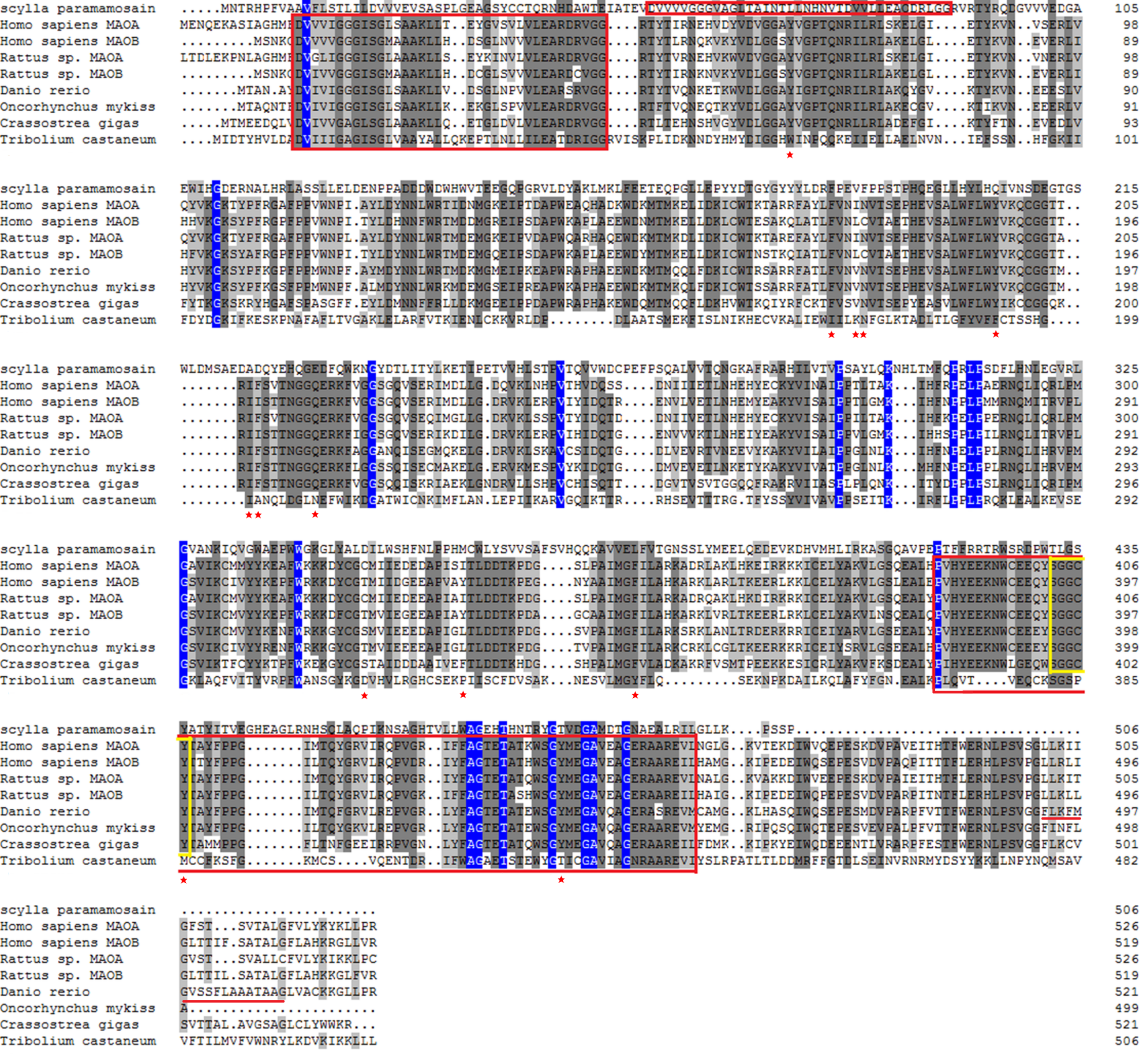
Alignment of the amino acid sequence of *SpAMO* with MAO sequences of other species. The GenBank accession number for MAOs are *Homo sapiens* MAO-A and MAO-B (M68840 and M69177), *Rattus sp*. MAO-A and MAOB (D00688 and M23601), *Danio rerio* MAO (AAO16681.3), *Oncorhynchus mykiss* MAO (AAA64302.1), *Crassostrea gigas* MAO (CAD89351.1), and *Tribolium castaneum* MAO (XP_015839656.1). The red box represents the FAD-binding domain. The FAD-binding pentapeptide is in the yellow box. The C-terminal putative transmembrane domain of *Danio rerio* is red underlined. The amino acids lining the substrate-binding site in *Homo sapiens* MAO-B are marked with a red asterisk.

Two highly conserved regions, a FAD-binding domain and a flavin-containing amine oxidoreductase domain, are described in all known MAO sequences. These domains also appeared to be highly conserved in *SpAMO*, where they were located at the positions of 53–89, 61–169, and 208–498 aa, respectively. In the NCBI alignment, three conserved domains—PLN0568 (polyamine oxidase), YobN (monoamine oxidase) and proto_IX_ox (protoporphyrinogen oxidase)—were identified in *SpAMO* and were located at the positions of 64–498, 64–502, and 77–133 aa, respectively. The flavin-containing amine oxidoreductase domain is conserved among many amine oxidases, including maze polyamine oxidase (PAO) and various flavin-containing MAOs. The conserved domain proto_IX_ox encodes protoporphyrinogen oxidase; this enzyme oxidizes protoporphyrinogen IX to protoporphyrin IX, a precursor of haeme and chlorophyll. The red asterisk in Fig 2 shows the essential amino acids for the substrate-binding sites of *Homo sapiens* MAO-B and *Rattus sp*. MAO-A[16,52]. These essential amino acids sites are Y60, F168, V171, N172, Y188, I198, F199, Q206, T314, L326, F343, Y398 and Y435, respectively. Based on the match information of the sequences, we observed that only residues F168 and Y398 of *Homo sapiens* MAO-B were coincident with *SpAMO*. Through the comparison of mammals and fishes, we found that only amino acids of Y60, F168, I198, Q206, F343, Y398, and Y435 of *Homo sapiens* MAO-B and *Rattus sp*. MAO-B were uniform with those of *Danio rerio* MAO and *Oncorhynchus mykiss* MAO. It was also consistent with *Homo sapiens* MAO-A and *Rattus sp*. MAO-A.

The homology between the deduced amino acid sequence of *SpAMO* and the sequences of other enzymes was analysed with the NCBI BLAST algorithm. It was found that there was relatively high homology between *SpAMO* and SMO genes from other species. Most of the SMO sequences were from insects; *SpAMO* shared 31% similarity with a *Helicoverpa armigera* SMO (XP_021195874.1), 29% similarity with a *Pieris rapae* SMO (XP_022126040.1), 28% similarity with a *Camponotus floridanus* SMO (EFN70212.1), and 27% similarity with an *Anoplophora glabripennis* SMO (XP_018567631.1). The similarities between *SpAMO* and SMOs from *Cyprinus carpio* (KTG11709.1) and *Homo sapiens* (AAH00669.1) were 19% and 17%, respectively. The amino acid sequences of the MAO genes of multiple species were downloaded from NCBI and compared with each other by DNAMAN software, and then the conservativeness of different domains was compared. The deduced amino acid sequences of *SpAMO* and MAOs from other species are aligned in Fig 2. Under normal circumstances, the sequence identity of nucleotides is lower than that of amino acids because of the existence of mutations. Thus, for an analysis of potential protein function, a protein sequence analysis was performed. The results of an amino acid sequence alignment showed that the *SpAMO* amino acid sequence was significantly different from the MAO sequences of other species. *SpAMO* displayed 11.8%, 12.7%, 12.0%, 12.4%, 11.1%, 11.8%, 13.0% and 10.8% identity with *Homo sapiens* MAO-A, *Homo sapiens* MAO-B, *Rattus sp*. MAO-A, *Rattus sp*. MAO-B, *Danio rerio* MAO, *Oncorhynchus mykiss* MAO, *Crassostrea gigas* MAO and *Tribolium castaneum* MAO, respectively. We found that *SpAMO* shared lower similarity with other MAOs, and, in general, the similarity between *SpAMO* and MAO was lower than that between *SpAMO* and SMO. Multiple sequence alignment showed that *SpAMO* shared the highest similarity with MAO of *C*. *gigas* (13.0%).

**Fig 3:**
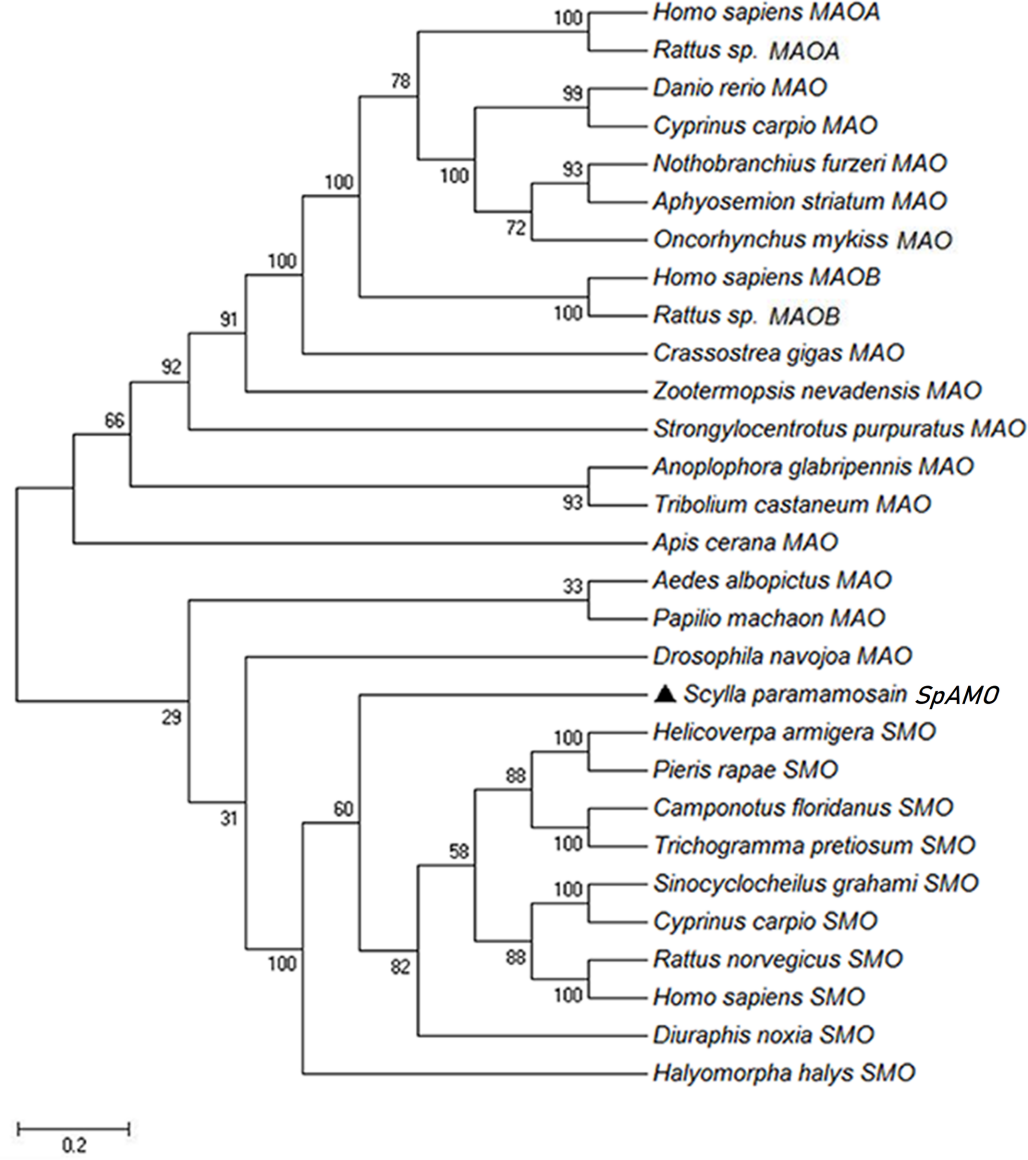
Evolutionary tree of MAO and SMO. The tree was constructed using the neighbour-joining algorithm in the MEGA 5.1 program based on multiple sequence alignment by Clustal W, and the reliability of the branching was tested using bootstrap resampling (1000 pseudo-replicates). The scale bar corresponds to the estimated number of amino acid substitutions per site. The genes used and their GenBank accession numbers are as follows: *Homo sapiens* MAO-A and MAO-B (M68840 and M69177), *Rattus sp*. MAO-A and MAO-B (D00688 and M23601), *Drosophila navojoa* MAO (XP_017966793.1), *Danio rerio* MAO (AAO16681.3), *Nothobranchius furzeri* MAO (SBP45138.1), *Aphyosemion striatum* MAO (SBP07926.1), *Oncorhynchus mykiss* MAO (AAA64302.1), *Cyprinus carpio* MAO (BAH02786.1), *Zootermopsis nevadensis* MAO (KDR09121.1), *Pieris rapae* MAO (XP_022115701.1), *Aedes albopictus* MAO (XP_019931073.1), *Anoplophora glabripennis* MAO (XP_018561137.1), *Tribolium castaneum* MAO (XP_015839656.1), *Papilio machaon* MAO (XP_014364998.1), *Hyalella azteca* MAO (XP_018020258.1), *Strongylocentrotus purpuratus* MAO (XP_003726162.2), *Crassostrea gigas* MAO (CAD89351.1), *Apis cerana* MAO (XP_016915250.1), *Helicoverpa armigera* SMO (XP_021195874.1), *Pieris rapae* SMO (XP_022126040.1), *Camponotus floridanus* SMO (EFN70212.1), *Sinocyclocheilus grahami* SMO (XP_016149021.1), *Cyprinus carpio* SMO (KTG11709.1), *Trichogramma pretiosum* SMO (XP_014236465.1), *Rattus norvegicus* SMO (NP_001128326.1), *Homo sapiens* SMO (AAH00669.1), *Diuraphis noxia* SMO (XP_015369715.1), and *Halyomorpha halys* SMO (XP_014291094.1).

The amino acid sequences of MAO and SMO proteins in mammals, fish and insects were downloaded from the NCBI protein database, and the predicted amino acid sequence of *SpAMO* was compared with these amino acid sequences using MEGA 5.1 software. The two-parameter model was used to establish a neighbour-joining phylogenetic tree (Fig 3). The evolutionary tree of amine oxidase was divided into MAO and SMO, and the two amine oxidases were clustered into mammalian, fish and insect groups. The phylogenetic tree further showed two branches for vertebrates and invertebrates. It is worth noting that the mud crab *SpAMO* clustered with the SMO genes and formed one invertebrate group with the insects, indicating that the genetic distance between these genes was small.

**Fig 4:**
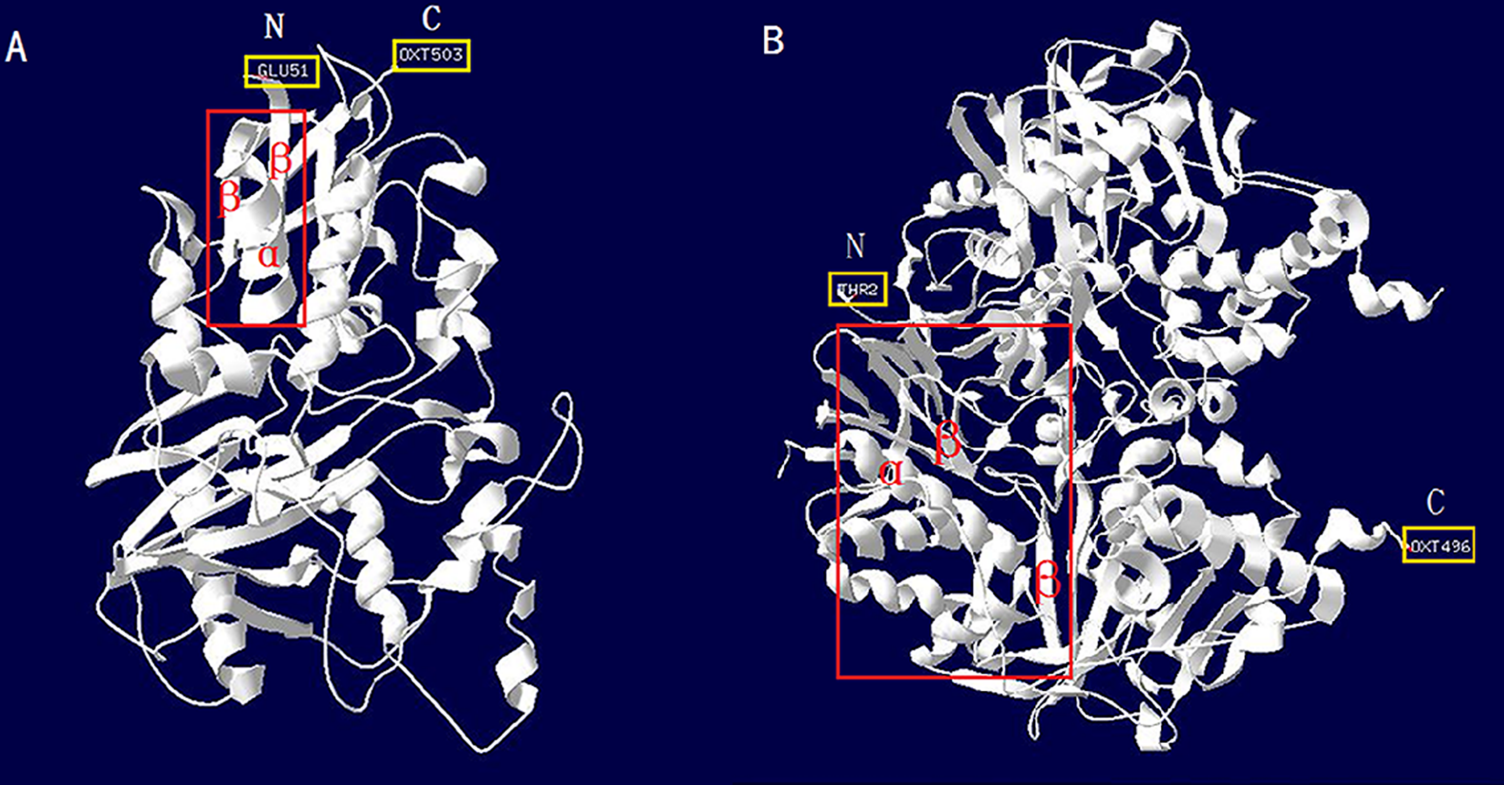
**Three-dimensional structure models of *SpAMO* (A) and MAO (B) from *Scylla paramamosain* and *Danio rerio*, respectively, established based on the SWISS-MODEL server.** “N” indicates the N-terminal residue; “C” indicates the C-terminal residue. The red boxes (A and B) represent the FAD-binding domain (located at 53–89 aa and 381–452 aa in A and B, respectively), which includes one alpha helix and two extended strands.

The putative secondary structure was composed of 25.9% alpha helices, 17.0% extended strands and 57.1% random coils, suggesting that random coils are the major component in *SpAMO*. A three-dimensional *SpAMO* structure (Fig 4A) with 28.25% identity to the murine peroxisomal N(1)-acetyl-spermine/spermidine oxidase (PDB accession No. 5mbx.1.A) was obtained by searching the Protein Data Bank (PDB) with this programme. The comparative analysis revealed that although there were some differences between the *SpAMO* protein sequence and MAO sequences of other species, the spatial structure of the FAD-binding domain was highly conserved among the various species. The predicted 3-D model of MAO (GenBank accession number: AAO16681.3) from *Danio rerio* shows the highly conserved structure of the FAD-binding domain (Fig 4B). The FAD-binding domain is located at the positions 53–89 and 381–452 of the amino acid sequence (Fig 4A and 4B). All FAD-binding domains have one alpha helix and two extended strand structures in that part of the protein.

### 3.2 Quercetin down-regulated *SpAMO* mRNA expression *in vivo*

**Fig 5:**
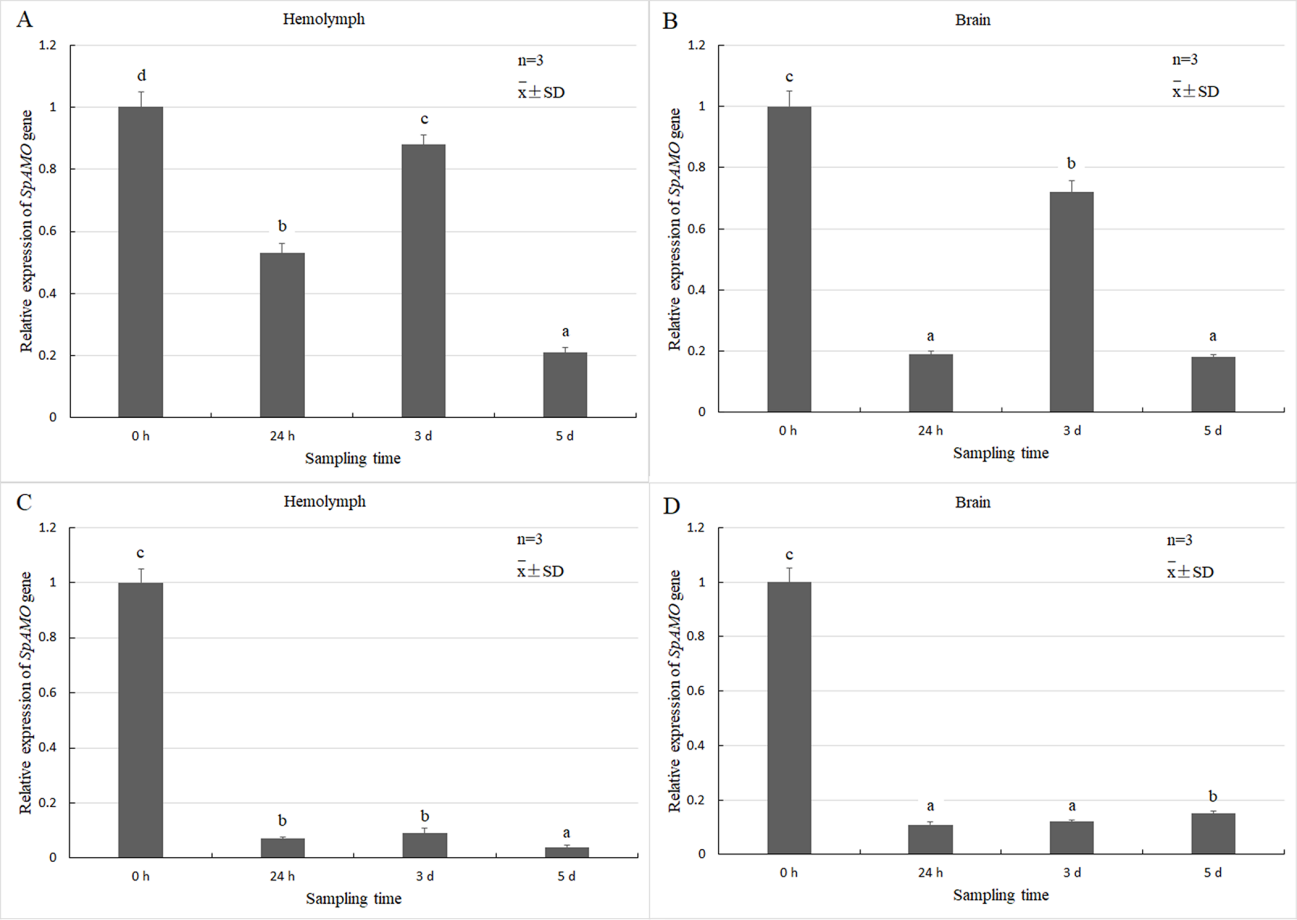
The relative expression levels of *SpAMO* from *Scylla paramamosain* after different doses of quercetin. Panels A and B show the results for the 5 mg/kg/d dose, while panels C and D show the results for the 50 mg/kg/d dose. The *SpAMO* mRNA expression was normalized to the 18S rRNA transcript level. Data are shown as the means ± SD (standard deviation) of three replicates. Means with the same letters are not statistically different; means with different letters are statistically different (*P*<0.05). The y-axis represents the ratio of the expression levels of *SpAMO*/*18S rRNA* mRNA.

Temporal expression of the *SpAMO* gene was investigated using qRT-PCR after a quercetin challenge (Fig 5). There was no difference in the *SpAMO* gene expression in haemolymph and brain tissue in the absence of quercetin. When crabs were treated with 5 mg/kg/d quercetin, the expression level of the *SpAMO* gene in haemolymph and brain tissues tended to fluctuate, first being down-regulated, then up-regulated, and then down-regulated again (Fig 5A and 5B). The expression level of the *SpAMO* gene in haemolymph and brain tissue decreased to 50% and 20% of the original level, respectively, by 24 h after treatment. Expression was up-regulated in both tissues on the 3rd day. By the 5th day, the expression was down-regulated compared to 24 h levels in haemolymph and had returned to 24 h levels in brain tissue. Furthermore, the down-regulation effect of quercetin on brain tissue was approximately 2.5-fold greater than its effect on haemolymph in the first 24 h after treatment. When the dose of quercetin was 50 mg/kg/d, the down-regulation effect was more pronounced, and the expression level of the *SpAMO* gene in haemolymph and brain tissues was reduced to approximately 10% of control levels (Fig 5C and 5D). The expression profiles of *SpAMO* was maintained at a stable and low level, and the down-regulation in haemolymph was more evident.

### 3.3 Enzymatic properties of *SpAMO*

**Fig 6:**
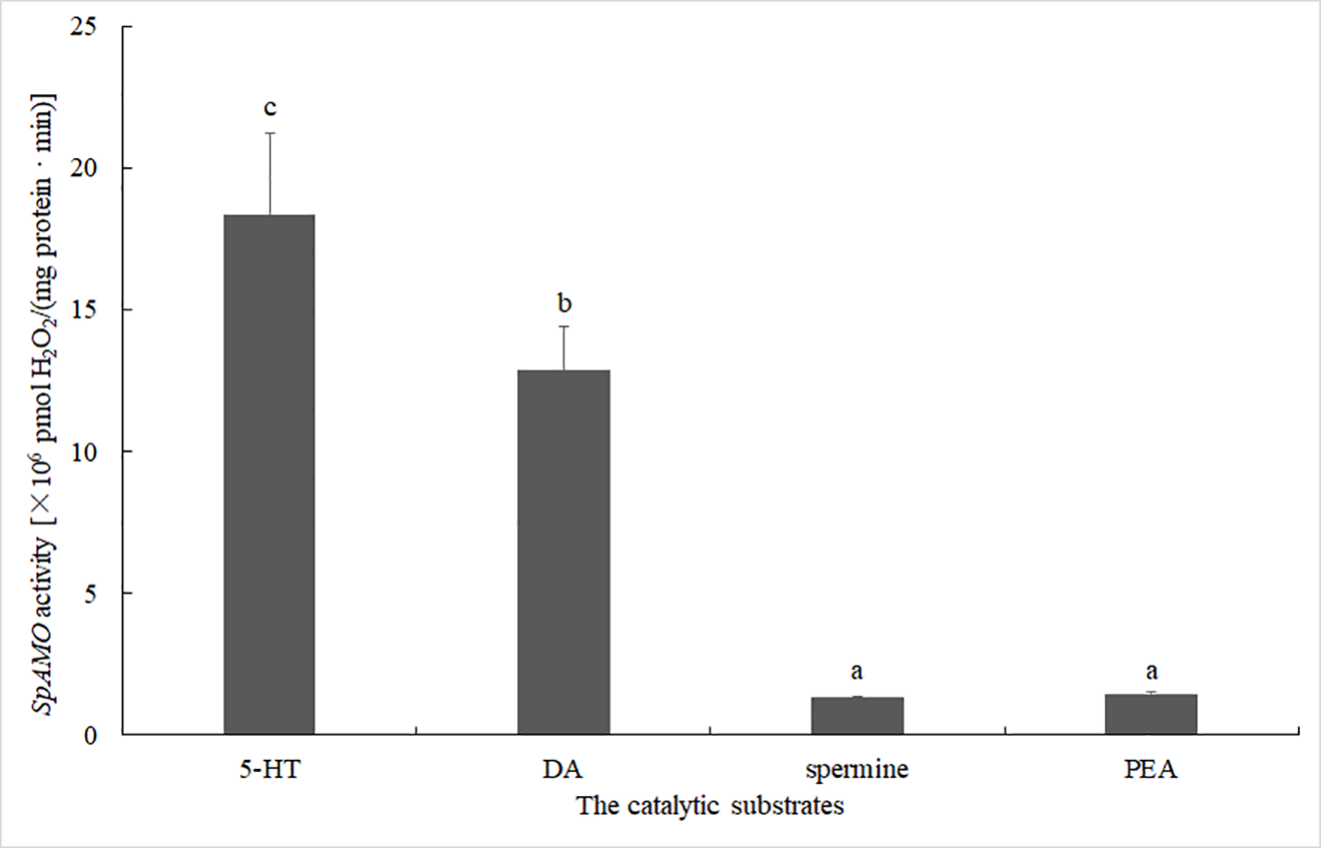
The activity values of SpAMO on four different biogenic amine substrates. *SpAMO* activity was measured using 5-HT, DA, spermine and PEA as substrates (50 μL), and an enzyme activity unit was defined as pmol H_2_O_2_/mg protein·min. Means with the same letters are not statistically different; means with different letters are statistically different (*P*<0.05).

To determine the specificity of the *SpAMO* catalysing substrate, four amines were selected for experiments, including 5-HT, DA, spermine and PEA. In mammals and other higher animals, 5-HT and DA are primarily deaminated by MAO, and spermine is degraded by SMO. Under the adopted experimental conditions *in vitro*, the results showed that *SpAMO* and all four substrates could produce reactive oxygen species (H_2_O_2_) by oxidative deamination (Fig 6). However, oxidative deamination of 5-HT and DA by *SpAMO* was significantly stronger than that of spermine and PEA. The enzyme activity of *SpAMO* against 5-HT and DA was much higher than its activity against spermine and PEA, and the enzyme activity against 5-HT was slightly higher than the activity against DA *in vitro*. Through fluorescence counting, the enzyme activity values of *SpAMO* were measured for the four substrates. *SpAMO* displayed values of 18.4, 12.8, 1.3, and 1.4 for 5-HT, DA, spermine and PEA, respectively. Thus, it could be concluded that *SpAMO* mainly catalyses 5-HT and DA in *S*. *paramamosain*.

## 4. Discussion

As mentioned above, the *SpAMO* nucleotide and deduced amino acid sequences showed low identity with MAO sequences from mammals, fish, insects and molluscs. The results of multiple sequence alignment revealed considerable differences in primary structure between *SpAMO* and MAO. In particular, *SpAMO* is more similar to SMO in terms of primary structures such as nucleotides and amino acids sequences. Related studies on MAO have been focused on mammals and other vertebrates, and MAO-A and MAO-B have been characterized based on their substrate specificities and inhibitor sensitivities [53–55]. Analysis of the secondary and tertiary structures of these two enzymes, as well as research with chimeric enzymes and site-directed mutagenesis, has made outstanding progress in elucidating the recognition mechanisms of MAO-A and MAO-B for different substrates [52,56–59]. A large number of reports on MAO mainly focus on mammals [56,60–62], not non-mammalian vertebrates such as fish and reptiles, let alone crustaceans. The currently available evidence suggests that there is a single and novel MAO form in teleosts that is more similar to MAO-A than to MAO-B [45]. Such single and novel forms of MAO have also been found to exist in perch [63], pike [64], rainbow trout [65], zebrafish [45] and carp [46]. The single form of MAO in fish may be the evolutionary precursor of both MAO forms (MAO-A and MAO-B) in mammals [46]. Interestingly, a new MAO type C-like dehydratase gene (MAO-C), which supplies R-3-hydroxyacyl-CoA from the fatty acid oxidation pathway to the polyhydroxyalkanoate (PHA) biosynthetic pathway, was identified in *Phytophthora capsici* [66]. In summary, whether there is a novel MAO with a similar crystal structure, similar substance-binding features, and similar inhibitor sensitivity in crustaceans such as *S*. *paramamosain* needs further exploration. At the same time, data regarding MAO genes in other crustaceans is also critical.

The deduced amino acid sequence and secondary structure prediction studies have shown that the flavin-binding domain is highly conserved in both the carboxyl-terminal and the amino-terminal regions of *SpAMO*. Overall, almost all reported MAOs have three conserved domains: the flavin-binding domain, the substrate-binding domain and the membrane-binding domain that anchors the enzyme to the outer mitochondrial membrane [45]. It has been reported that nine cysteine residues exist in the deduced amino acid sequence of carp MAO and in both forms of human liver MAO [46,67]. Sugimoto et al. [46] also confirmed the presence of a pentapeptide (Ser-Gly-Gly-Cys-Tyr) in carp MAO that may bind to FAD. The cysteine residues of thioether link to the flavin ring, and the cofactor FAD binds to this pentapeptide. In addition, a corresponding region of the α-helix motif in the mitochondrial membrane that is used to insert the protein into a fragment with 18 amino acid residues was also found in carp and zebrafish MAO [45–46,65] but it hasn’t been found in trout MAO [65]. This C-terminal putative transmembrane domain of zebrafish MAO is also not found in *SpAMO*. This transmembrane α-helix domain does not seem to be conserved between zebrafish and carp, neither of which is conserved with respect to mammal MAOs sequence. Studies have found that in mammals, the sequence of C-terminal transmembrane α-helix is not conserved between the two forms of MAO-A and MAO-B, but it is conserved among the MAO-A or MAO-B from different species [45]. The 18 essential amino acids for substrate-binding sites of human MAO-B listed in Fig 2 are replaced by other amino acid residues at different positions in different species. For example, leucine 171 was replaced by valine in fish MAO and *SpAMO* and replaced by isoleucine in MAO-A. The effect of amino acids substitution probably is not significant because these three amino acids have similar properties. All three amino acids mentioned above belong to hydrophobic non-polar amino acids. Hydrophilic polar cysteine at position 172 and the hydrophobic non-polar aliphatic residue isoleucine at position 199 in human MAO-B were replaced in *SpAMO* by a non-polar, aromatic phenylalanine and by non-polar aliphatic alanine, respectively. In fish MAO and mammal MAO-A, the amino acids at these two positions were replaced by more similar polar aliphatic asparagine and non-polar aromatic amino acid phenylalanine. 206 glutamine, 343 phenylalanine, and 435 tyrosine located in human MAO-B were replaced by glutamic acid, leucine, and threonine in *SpAMO*, respectively. Although there are different degrees of substitution of the amino acids at the substrate-binding site in different species, in most cases the binding properties of the substrate are not affected. One of the main reasons is the presence of properties similar to amino acids. Furthermore, in addition to the residues lining the substrate-binding site, other amino acid residues could also be crucial in the molecule’s conformation [45]. Moreover, for human MAO-B and rat MAO-A, there is a three-dimensional model based on the crystal structure [16]. Because of such research, today we can not only compare the molecular features of entire sequences but also check the substrate and inhibitor preferences of conserved sequences. The overall folding structures of human MAO-B and rat MAO-A appear to be very similar but show a different conformation in the critical substrate-binding site. According to the human MAO-B three-dimensional model, the substrate-binding domain appears to be divided into two chambers of varying sizes, and the substrate enters the substrate-binding cavity from the smaller channel cavity [16]. The movement of the channel cavity causes the substrate to diffuse into a gate consisting of four residues (Y326, I199, L171, F168); the gate then enters the substrate-binding chamber by transient movement. The structure of rat MAO-A does not reveal any substrate entrance pathway like that of human MAO-B, suggesting that the rat MAO-A substrate enters into the active site by a conformational change of the molecule [52].

Quercetin injection experiments showed that quercetin can effectively down-regulate the transcription of *SpAMO*. *SpAMO* gene expression decreased first and then increased when the quercetin dose was at a low concentration (5 mg/kg/d). This may be explained by a compensatory mechanism which compensates for the loss of enzyme activity by producing more enzymes. When the concentration was high (50 mg/kg/d), *SpAMO* remained at a stable and low mRNA level. The reason for this may be that the effect of high concentrations of quercetin exceeds the threshold for self-regulation of mud crabs. Quercetin is a flavonoid widely distributed in plant foods and herbal medicine and is mainly present as glycosides in plants. Currently, studies suggested that quercetin and other structurally related flavonoids exert antidepressant-like effects in rodent models of depression, and these effects were achieved mostly through acting as the protein inhibitor [68–70]. For example, *in vitro* studies have shown that quercetin has a considerable inhibitory effect on MAO-A activity in mitochondrial fractions obtained from mouse brain [71–72]. Quercetin exerts an inhibitory effect on the mitochondrial MAO-A reaction in the mouse brain by reducing the deamination product of 5-HT [72]. In a study on human neuroblastoma SH-SY5Y cells, it was also found that the flavonol quercetin could localize to the outer mitochondrial membrane and attenuate MAO-A activity directly in neuronal cells [73]. In particular, the researchers used docking simulation studies to show that quercetin has a high affinity for the MAO-A protein and to provide the lowest interaction energy configuration of the quercetin and MAO-A complex [71]. This interesting phenomenon deserves our attention because quercetin significantly down-regulated the expression of *SpAMO* in this study. What we found was different from the previous research which regarded quercetin as a protein inhibitor. Behind this phenomenon must be a complex regulatory network that affects the transcription level of the *SpAMO* gene in an indirect way. Studies have shown that quercetin and its derivatives can use microRNA as a molecular target, and microRNA is involved in post-transcriptional gene silencing and regulates expression of genes involved in physiological processes such as development, proliferation, metabolism and inflammation [74]. As a transcriptional repressor, mammalian microRNA recognizes and binds to the 3'-UTR of the target gene by complementary pairing of complete or incomplete sequences, which destabilizes the target mRNA to reduce protein synthesis [75–78]. In addition, small interfering RNA (siRNA) is an initiator of RNA interference, which can deactivate the complementary target mRNA [79]. In a word, the regulatory mechanisms involved in this phenomenon and the relation and the regularity between them are worthy of our in-depth study.

The enzymatic activity experiment for *SpAMO* showed that the catalytic activity of *SpAMO* on 5-HT and DA was much higher than its activity on spermine and PEA. The PAO of yeast is called Fms1, which can oxidize spermine, N-acetyl spermine and N-acetyl spermidine [80–81]. However, in vertebrates, two different enzymes, SMO and acetylpolyamine oxidase (APAO), specifically catalyse the oxidation of spermine and acetylated polyamine, respectively. The yeast PAO can catalyse the oxidation of acetylated and non-acetylated polyamines, and in vertebrates, these two functions are undertaken by SMO and APAO, respectively, so it was speculated that the front part of the enzyme may be the ancestor of these two enzymes, having replicated and produced the parallel homologous genes SMO and APAO in vertebrates [82]. MAO-A and SMO belong to the flavoprotein family; the difference between them is that MAO-A is mainly involved in monoamine neurotransmitter metabolism, while SMO mainly participates in polyamine metabolism. Another thing which should be mentioned is the oxidation of serotonin and dopamine were proved through an indirect measurement of H_2_O_2_ generation. Determination of polyamine oxidase activities using H_2_O_2_ generation was successfully used [51]. The difference in the inhibitory effect of quercetin on MAO-A in different literatures may be method bias [83]. Moreover, the effect of quercetin on *SpAMO* and MAO-A may not be equal. Some researchers think that assays in which the evaluation of inhibition on MAO is only based on oxidation of peroxidase substrates may lead to erroneous results as seen here for some phenolic compounds. These compounds are antioxidants that may interfere with peroxidase assays [83]. Therefore, this enzyme activity assessment should be confirmed by a direct method like HPLC (MS) in the further studies.

In this study, we characterized a novel amine oxidase (*SpAMO*) in *S*. *paramamosain*. The complete cDNA sequence encoding this flavoprotein enzyme is involved in monoamine neurotransmitter metabolism. *SpAMO* demonstrated the molecular characteristics of SMO in bioinformatics studies such as nucleotide and amino acid sequence analysis, multiple sequence alignment, phylogenetic relationship determination and three-dimensional structure prediction. However, the quercetin test and substrate specificity analyses suggested that *SpAMO* has the activity of MAO-A. Therefore, these findings seem to support the hypothesis that *SpAMO* may be a special form of amine oxidase that shares the biological functions of SMO and MAO-A in *S*. *paramamosain*.

## Supporting information

S1 File Secondary structure of *SpAMO* protein. (PDF)

S1 Fig SDS-PAGE analysis of the recombinant protein *SpAMO*. M: protein marker. Lane 1: the negative control. Lane 2: inclusion body of *E*. *coli* BL21 (DE3) strain with recombinant vector *SpAMO/pCold I*. Lane 3: supernatant of *E*. *coli* BL21 (DE3) strain with recombinant vector *SpAMO/pCold I*.
(TIF)

S2 Fig SDS-PAGE analysis of the recombinant protein *SpAMO* after Ni-NTA purification. M: protein marker. Lane 1 and 2: elution products (5 μL).
(TIF)
