# Supplementary figures and images for: An amine oxidase gene from mud crab, Scylla paramamosain, regulates the neurotransmitters serotonin and dopamine in vitro
Source: PLoS One. 2018 Sep 24;13(9):e0204325. doi: 10.1371/journal.pone.0204325 (PMC6152983; doi:10.1371/journal.pone.0204325)

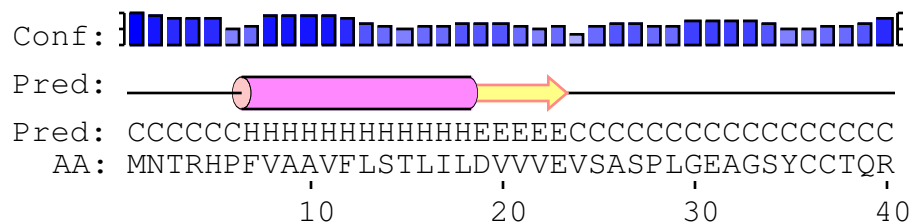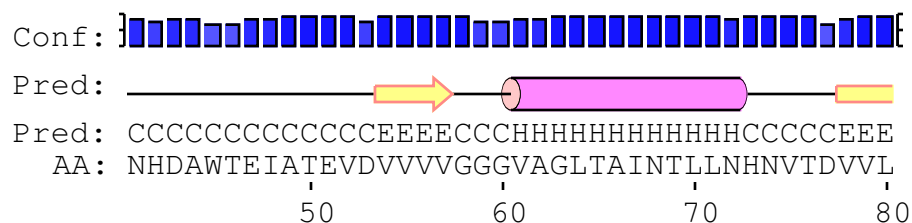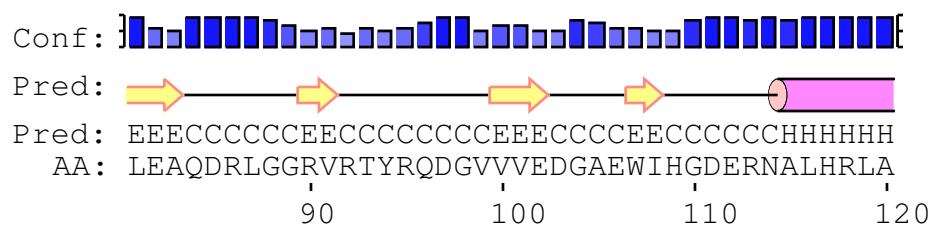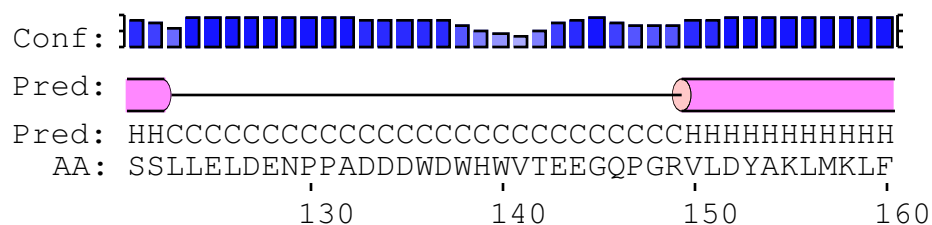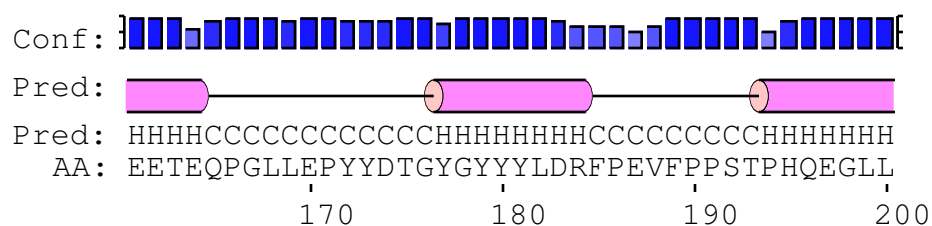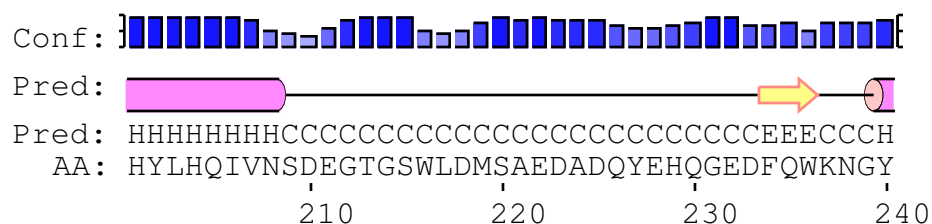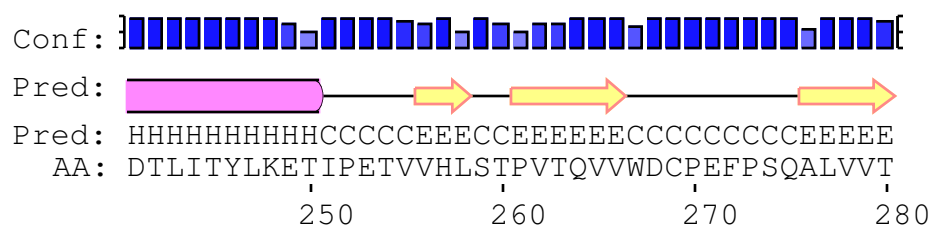

Supplement: S1 File — (PDF) [file pone.0204325.s001.pdf]

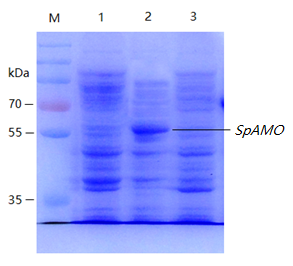

Supplement: S1 Fig — M: protein marker. Lane 1: the negative control. Lane 2: inclusion body of E. coli BL21 (DE3) strain with recombinant vector SpAMO/pCold I. Lane 3: supernatant of E. coli BL21 (DE3) strain with recombinant vector SpAMO/pCold I. (TIF) [file pone.0204325.s002.tif]

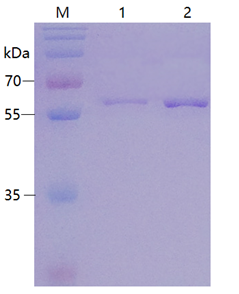

Supplement: S2 Fig — M: protein marker. Lane 1 and 2: elution products (5 μL). (TIF) [file pone.0204325.s003.tif]
